# Seasonal Prevalence and Molecular Identification of Thermophilic *Campylobacter* from Chicken, Cattle, and Respective Drinking Water in Kajiado County, Kenya

**DOI:** 10.1155/2022/1526641

**Published:** 2022-09-27

**Authors:** Daniel W. Wanja, Paul G. Mbuthia, Gabriel O. Aboge, Lilly C. Bebora

**Affiliations:** ^1^University of Nairobi, Faculty of Veterinary Medicine, Department of Veterinary Pathology Microbiology and Parasitology, P. O. Box 29053-00625, Kangemi, Nairobi, Kenya; ^2^Animal Health and Industry Training Institute (AHITI) Kabete, P. O. Box 29040-00625, Kangemi, Nairobi, Kenya; ^3^Department of Animal Science, Chuka University, P.O Box 109-00625, Chuka, Kenya; ^4^University of Nairobi, Faculty of Veterinary Medicine, Department of Public Health Pharmacology and Toxicology, P.O. Box 29053-00625, Kangemi, Nairobi, Kenya

## Abstract

Thermophilic *Campylobacter* species are a leading cause of human gastroenteritis throughout the world and have been implicated in reproductive disorders (abortion), mastitis, enteritis, and/or diarrhoea in livestock. A cross-sectional survey was conducted in Kajiado County to determine prevalence, seasonality, and molecular detection of thermophilic *Campylobacter* species (with emphasis on *C*. *jejuni*, *C*. *coli*, and other thermophilic *Campylobacter* species) in chicken, cattle, and respective pooled drinking water. A total of 457 samples comprising 265 cattle rectal swabs, 142 chicken cloacal swabs, and 50 trough water samples were collected from 55 randomly selected smallholder farms. Individual samples were subjected to standard techniques for isolation and biochemical tests, followed by singleplex polymerase chain reaction (sPCR) assays for identification and confirmation of genus and species. Overall, thermophilic *Campylobacter* prevalence was 35.4% (95% confidence interval (95% CI) = 31.0–39.8), with *C*. *jejuni* dominating at 55.6% (95% CI = 47.9–63.3%) over *C. coli* in all sample types. The highest thermophilic *Campylobacter* prevalence was observed in cloacal swabs of live chicken at 44.4% (95% CI = 36.2–52.6%), followed by rectal swabs from live cattle at 30.9% (95% CI = 25.3–36.5%). Water samples from cattle drinkers/trough were found to be contaminated at 34% (95% CI = 20.9–47.1%). The isolation rate was higher in cattle under the confinement system (44.3%) (95% CI = 36.1–52.5%) than in those under the free-roaming grazing system. Thermophilic *Campylobacter* species were isolated in both seasons, with higher prevalence (39.8% (95% CI = 33.6–45.9)) recorded during rainy and cold season in all sample types except for water. There was significant (*P* < 0.05) association between season and thermophilic *Campylobacter* occurrence, even though there were no statistical differences in the prevalence values across the two seasons. Results of this study demonstrate that cattle, chicken, and respective drinking water harbour potentially pathogenic thermophilic campylobacters, with *C. jejuni* being widely distributed among farms. It is possible that seasonal variations and cattle confinement result in differences in thermophilic *Campylobacter* carriage. Further epidemiological and phylogenetic studies comparing distribution of thermophilic *Campylobacter* spp. isolates in livestock, environmental, and human samples are recommended to establish source attribution to reduce the impact of resultant diseases for the wellbeing of public and livestock.

## 1. Introduction

Campylobacters normally exist as small (0.2–0.8 *µ*m × 0.3–5 *µ*m), Gram-negative, motile, spirally curved/spiral rods but can occasionally exist as long-form (8 *µ*m) spiral rods. However, under unfavourable culture conditions, the spiral rods degenerate to coccoid forms [1]. Like other bacteria, the coccoid forms were firstly believed to be a viable but nonculturable form with ability to survive in unfavourable circumstances. Nonetheless, subsequent research on closely related bacterium *Helicobacter pylori* hinted that the coccoid forms are nonviable and degenerative forms [2]. To date, the issue on whether the coccoid forms are viable and infective has been exceedingly contentious ([2]. However, Frirdich et al. [3] demonstrated that evolution to coccoid forms resulted in variations in pathogenicity and/or virulence. Frirdich et al. [3] further reported that coccoid forms of *C. jejuni* are nonmotile and noninfectious, with minimal invasion and adhesion of epithelial cells and an inability to stimulate neutrophil chemoattractant and/or interleukin-8 (IL-8).

Thermophilic campylobacters, particularly *C. jejuni* and *C. coli*, are of most clinical relevance and are also most frequently encountered. Other species *C*. *concisus*, *C. lari*, *C*. *upsaliensis*, and *C*. *ureolyticus* are described as “emerging *Campylobacter* species” and have been underrated as causal agents of human gastroenteritis because of biases in the existing identification methods [4, 5]. Thermotolerant campylobacters are presumed to be commensals in birds and insignificant for avian health, with reports indicating that the bacterium may colonise the palatine lymphoid tissues and the crops in broilers, with higher colonisation in intensive systems [1]. The organisms have been reported to cause pathology in other farm animals including *C*. *jejuni* subsp. *jejuni* (enteritis and abortion in cattle and shoats), *C*. *coli*, and *C*. *mucosalis* (enteritis in pigs); *C*. *upsaliensis* and *C*. *helveticus* (enteritis in dogs and cats); *C*. *hyointestinalis* subsp. *hyointestinalis* (enteritis in cattle and pig); and *C*. *sputorum* (ovine abortions) [1]. The bacterium is commonly associated with gastroenteritis and systemic illness in humans. As such, campylobacters are important targets for animal and human health research because of their zoonotic potential, wide variety of reservoir hosts, and environmental persistence and survival (especially in water) [6].

In Kenya, several studies have focused on chicken and chicken products as the major reservoir of *Campylobacter* infection [7–12]. Most studies have incriminated poultry and poultry products for the increasing cases of human campylobacteriosis worldwide. It is, however, surprising that a rising trend in human *Campylobacter* infection has been reported in Scandinavian countries, where thermophilic *Campylobacter* carriage in poultry flocks is low [13]. This therefore suggests that there are other likely sources of this bacterium. Nevertheless, studies carried out elsewhere on source ascription have shown that, among other livestock, cattle are common carriers of *Campylobacter jejuni* and could be a probable source of human campylobacteriosis [14, 15]. However, minimal information is available on thermophilic *Campylobacter* epidemiology in cattle in Kenya. In particular, the effect of cattle housing on colonisation of thermophilic campylobacters remains ill defined.

This is further compounded by lack of surveillance data on incidence of thermophilic *Campylobacter* infections as most laboratories do not routinely test for the bacterium; it is seen as a silent threat. This study has investigated the epidemiology of *Campylobacter* colonisation in cattle so as to elucidate its role in human infections. Literature search has indicated that only three studies have been conducted, in Kenya, on occurrence of thermotolerant *Campylobacter* in cattle and cattle products [7, 16, 17]. This necessitates the need to collect more recent information on the same through wide-range sampling in farm-fed and free-roaming cattle. Moreover, molecular characterisation has not been conducted in Kenya.

In order to further elucidate the epidemiology of thermophilic *Campylobacter* colonisation in cattle, other factors that may influence prevalence estimates need to be weighed in as well. Such factors include herd size, breed, age, animal husbandry practices, location, season, and closeness to infected poultry, just to mention a few. The study on seasonal effect on campylobacters carriage was based on the fact that *Campylobacter* colonisation in cattle and poultry in temperate and/or European countries has been widely described, with peak shedding occurring in either the winter or the summer [13, 18]. This effect has not been documented in tropical low- and middle-income countries, perhaps due to lack of studies in these settings [9] and/or inadequate surveillance. The seasonal peaks may coincide with levels in either faecal shedding in livestock or exposure to a common contamination source like pasture and water. Interestingly, upon environmental contamination, the bacterium may not survive long enough to colonise grazing cattle, apart from in water, where lengthy persistence of *Campylobacter* has been documented [19]. Therefore, contaminated water plays a major role as an environmental exposure for faecal-oral mode of disease spread in both livestock and humans. Although the relative contribution of water sources to *Campylobacter* infections in livestock is unclear, due to lack of data linking water to such infections in Kenya, studies conducted elsewhere have shown an association between water source and *C*. *jejuni* carriage [13, 20].

Given the above data gaps and the probable association among thermophilic *Campylobacter* spp. harboured by livestock (including poultry which are known reservoirs) and livestock environment (soil, untreated water, and manure) and human illness, more research on the same is justified. This study aimed at investigating the prevalence, seasonality, and molecular detection of thermophilic *Campylobacter* species (with emphasis on *C*. *jejuni*, *C. coli*, and other thermophilic *Campylobacter* spp.) in chicken, cattle, and pooled drinking water samples in Kajiado County, Kenya.

## 2. Materials and Methods

### 2.1. Ethical Consideration

This study was approved by Biosafety, Animal Use and Ethics Committee, Faculty of Veterinary Medicine, University of Nairobi, prior to commencement of the project (FVM BAUEC/2020/274). Verbal consent was sought from farm owners prior to sampling.

### 2.2. Study Area

**Figure 1:**
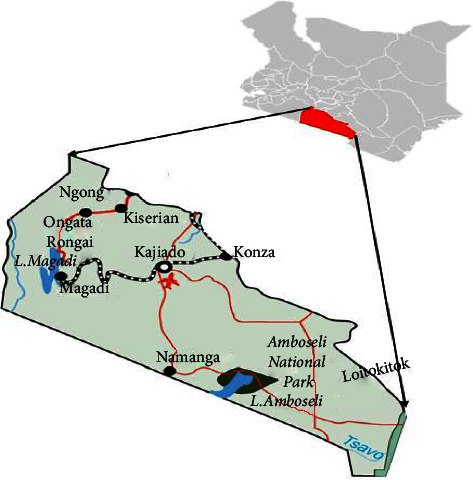
Map of Kajiado County and its location in Kenya (shaded red).

The study was conducted in Kajiado County, particularly in Kajiado North subcounty (areas of Ongata Rongai, Ngong), Kajiado West subcounty (Kiserian), and Kajiado East subcounty (Kitengela, Isinya, Mashuru) (Figure 1). Kajiado County neighbours Nairobi and spreads to Tanzania border further South and lies between latitude −2° 00′S and longitude 36° 52′E.

Kajiado County is preponderantly semiarid and is inhabited by Maasai ethnic group; however, persons from other regions in Kenya as well as foreigners have migrated there. In Kajiado, livestock production and marketing are the main economic activity, with about 70 percent of the people depending on livestock (including cattle, shoats, and poultry) and livestock products. The county has a distinct bimodal rainfall pattern: October to December's insubstantial and/or “short” rains and March to May's substantial and/or “long” rains. The months of January/February are usually hot and dry, while the months of June to August are cool and dry.

The county was purposively selected owing to its weather variability and vulnerability to climate change, especially rainfall fluctuations. The sampling sites were selected not only for their distinct variation in climatic patterns (for comparison) but also for their potential for livestock farming.

### 2.3. Study Design

A two-season-based cross-sectional-design study was carried out among smallholder cattle farms in Kajiado County, Kenya, between October 2020 and May 2022. The study involved collection of faecal samples (chicken cloacal swabs and cattle rectal swabs) and animal trough water samples in the enrolled farms.

### 2.4. Study Animals

Target population of interest consisted of cattle and chicken. Households rearing cattle and/or chicken were used as the sampling units, which in this study were considered as “study farms.” A list of farms (forming the sampling frame) were obtained from local livestock production offices.

### 2.5. Study Farms Enrollment and Size Determination

Farms were enrolled in this study based on the following criteria: (i) smallholder to medium farms raising multiple species (farms rearing cattle (≥200 cows) and other ruminants with or without chicken or farms keeping chicken (≥300 birds) with other ruminants with or without cattle) and (ii) free-roaming cattle under outdoor grazing and/or farm-fed cattle under zero grazing. In addition, farm owners' willingness and availability to participate were also considered. The minimum number of farms enrolled was guided by the formula *n*=*N*/1+*N*(*e*)^2^ [21], where “*N*” is the overall number of farmers in the county, while “*e*” is the error permitted for the population. Based on NAFIS [22], there are around 223 farms in Kajiado County. By applying the above formula at 12% error for *N* = 223, the number of farms enrolled was 50. However, with assistance from the local animal health providers, a total of 55 farms were recruited by simple randomisation. GPS coordinates for each farm were recorded.

### 2.6. Sample Size Determination and Sampling Strategy

The minimum number of samples was calculated by applying the formula by Thrusfield [23], *n*=*Z*^2^*P*(1 − *P*)/*d*^2^, where *n* is the sample size, “*Z*” is the *Z* statistic for 95% confidence (1.96), and “*P*” is anticipated *Campylobacter* prevalence, while “*d*” is the precision. The target sample size for cattle samples with presumed prevalence of 50% and a precision of 8% (0.08) was 150 animals. The expected prevalence for poultry samples was set at 69% [8] with a precision of 9% (0.09); this gave a sample size of 100 poultry samples. The number of individual faecal samples collected ranged from 1 to 7 per animal species, depending on herd/flock size per recruited farm. One unstirred water sample was collected from bovines' water troughs and/or watering points in each of the enrolled farms.

### 2.7. Sampling Plan and Sample Collection

All sampling events occurred between October 2020 and May 2022. The sampling scheme attempted to assess seasonal variations in occurrence of thermophilic campylobacters, such that sampling coincided with October–December and March–May (rain season) and with January-February and June–September (dry season). In addition, climatic data (minimum and maximum daily temperatures, relative humidity, and precipitation) spanning over the sampling period for two seasons were retrieved from local meteorological stations.

Proportional stratified sampling was conducted for each farm. Strata were based on livestock species (chicken versus cattle). Cattle derived samples were further stratified into either farm-fed/confined (zero-grazing) or free-roaming (outdoor grazing either settled or transhumant pastoral systems). Thus, the representative sampling plan entailed sampling of 407 faecal samples (comprising 265 cattle rectal swabs and 142 chicken cloacal swabs) and 50 surface water samples (from troughs and common watering points) from 55 households (described as “smallholder farms” in this study). Thus, a total of 457 samples distributed across two seasons (cold-wet and warm-dry season) were collected as tabulated in Table 1.

Cattle were restrained in a crush, and samples were collected by swabbing the rectoanal mucosa with cotton swabs. Swabbing was done aseptically using cotton-tipped swab sticks following a protocol described by Khaitsa et al. [24]. For chicken, they were restrained manually, with minimal force possible. Cloacal swabs were collected by introducing the whole tip of cotton swab into the cloaca and swabbing with two to four circular motions while applying gentle pressure against the mucosal surfaces. Immediately after swabbing, swabs were separately placed in bijou bottles containing Stuart's® transport medium (HiMedia®, Mumbai, India) and labeled accordingly. Unstirred animal trough water samples were collected aseptically into a 500 ml sodium thiosulphate sterilised bottle from sampled cattle pen(s). Samples from different study cattle pens (pens in which sampling was done) were pooled and taken as one sample. For cattle under outdoor grazing/transhumant, surface waters from watering points (dams and rivers) were sampled.

All samples (rectal swabs, cloacal swabs, and water samples) were labeled accordingly, placed in cooler boxes packed with ice packs, and immediately transferred to the microbiology laboratory in the department of Veterinary Pathology, Microbiology and Parasitology (VPMP) for *Campylobacter* culture within 3 hours.

### 2.8. Isolation, Culture Conditions, and Conventional Identification

For isolation, faecal samples were cultured using conventional culture methods optimised for the detection of thermophilic *Campylobacter* species. Briefly, swabs were loaded aseptically into 7 ml bijou bottles containing *Campylobacter* enrichment broth (Bolton broth without the addition of blood and selective supplement) (Oxoid). The bijou bottles were almost filled with the broths leaving a minimal headspace to prevent aerobiosis. After 24-hour incubation at 42°C, the broths were streaked aseptically onto modified charcoal-cefoperazone-deoxycholate agar (mCCDA) plates (incorporated with *Campylobacter* selective supplement) which were further incubated microaerobically at 42°C for 48 hours.

All water samples were processed by filtration method following a procedure described by Horman et al. [25] with slight modifications. Briefly, a 100 ml water sample was filtered through a sterile 0.45 *µ*m-pore-size cellulose nitrate filter membrane (CHMLAB®), and the filter was then placed in a universal bottle containing 20 ml of Bolton enrichment broth for *Campylobacter* without antibiotics. After 3 hours of incubation at 42°C, 0.2 ml of a selective supplement containing cefoperazone, vancomycin, trimethoprim, and cycloheximide (SR0167E, Oxoid®) was added. Incubation was continued microaerobically for further 24 hours at 42°C. After the selective enrichment phase, a 10^l^ portion of broth was spread onto the surface of a modified CCDA agar plate and incubated microaerobically at 42°C for 48 hours.

All the incubations of both broths and plates were done at 42°C, under microaerophilic conditions which were provided by burning candles in air-tight jars [26]. Afterwards, the plates were examined and the colony morphology on the plates was recorded. Pure cultures of the isolates were preserved in duplicate in Tryptose soya broth (HiMedia) supplemented with 30% (v/v) glycerol at –20°C awaiting PCR assays for confirmation and identification of thermophilic *Campylobacter* species.

Distinct colonies were subcultured to obtain pure colonies by restreaking onto blood agar plates (with selective supplement). Presumptive identification of the *Campylobacter* suspect colonies was done by culture characteristics (growth at 42°C), colony morphology, Gram staining characteristics, and biochemical reactions (oxidase, catalase, and hippurate hydrolysis reactions) following criterion given by Hendriksen et al. [27] and Markey et al. [28].

### 2.9. Extraction of Bacterial DNA and PCR Identification of the Genus *Campylobacter*

*Campylobacter* DNA was extracted using a heat lysis or boiling technique [29]. Briefly, previously preserved putative *Campylobacter* isolates were revived by subculturing on blood agar plates with selective supplement (BASs) at 42°C for 24 hours. The colonies (3–5) were then suspended in sterile distilled water in an Eppendorf tube. The resulting bacterial suspension was boiled in a water bath at 100°C for 30 minutes and then allowed to cool and later centrifuged at 15,000 rpm for 5 minutes. The supernatant containing DNA was transferred into a sterile Eppendorf tube, which was then preserved at −80°C.

A singleplex PCR assay was initially undertaken to detect 857 bp portion of 16 subunit ribosomal RNA (16S rRNA, a highly ubiquitous and extremely conserved region within the *Campylobacter* genome) gene for *Campylobacter* genus using forward-TCTAATGGCTTAACCATTAAAC and reverse-GGACGGTAACTAGTTTAGTATT primers [30]. The choice of 16S rRNA was largely due to the fact that it is ever-present in members of the genus *Campylobacter* and therefore ideal for primary identification. The primer sequences were subjected to BLAST analysis against the entire microbial genome database in GenBank (https://www.ncbi.nlm.nih.gov) to confirm primer specificity. A singleplex PCR assay was carried out in a 25 *µ*L reaction volume comprising 5 *µ*L of template DNA, 12.5 *µ*L master mix (New England Biolabs), 2 *µ*L of each of the forward primer and the reverse primer (Inqaba Biotechnologies, Pretoria, South Africa), and 3.5 *µ*L of nuclease-free water (BioConcept).

The PCR tubes (0.2 mL) containing amplification mixture were transferred to preheated 96-well thermal cycler (Bio-Rad T100™). The DNA was amplified using a program of initial heating at 95°C for 10 minutes followed by 30 cycles of denaturation at 94°C for 30 seconds, annealing at 56°C for 30 seconds, and extension at 72°C for 1 minute with a final extension of 72°C for 10 minutes.

The amplicons were verified by gel electrophoresis on ethidium bromide stained 1.5% agarose gel prepared by adding 3 grams of agarose (Cleaver Scientific Limited, Rugby, UK) to 200 ml tris acetate EDTA (10xTAE) buffer (Glentham Life Sciences, Corsham, UK) containing 1 *μ*g/ml ethidium bromide (Sigma, Dorset, UK) at 250 volts for 45 minutes and thereafter visualised on an ultraviolet transilluminator (UVP BioDoc-It™ imaging system, Cambridge, UK). Hyperladder IV (Bioline, London, UK) was used as the molecular weight marker and band positions were determined by eye using the molecular weight marker.

### 2.10. Polymerase Chain Reaction (PCR) Identification of *Campylobacter jejuni* and *C*. *coli*

All the PCR-confirmed thermophilic *Campylobacter* isolates were further subjected to a singleplex PCR assay to delineate the isolates to species level for the detection of *C. jejuni* and *C*. *coli*. The 600 bp fragment of hippurate hydrolase (*hipO*) gene of *C*. *jejuni* was amplified by PCR using the forward-TGATGGCTTCTTCGGATAG and reverse-CTAGCTTCGCATAATAACT primers [30]). In order to confirm *C. coli* isolates, the gene encoding siderophore transport protein (*ceuE*) was amplified with the forward-ATTGAAAATTGCTCCAACTATG and reverse-GATTTTATTATTTGTAGCAGCG primers [29]. The restricted fragments were amplified using the respective primer sequences and thermal cycler conditions were as described by Han [31]. *Campylobacter coli* (ATCC 33559) and *C. jejuni* (ATCC 33560) were used as positive controls, whereas sterile nuclease-free water was used as negative control. Amplicons were analysed by gel electrophoresis and then observed under ultraviolet light.

### 2.11. Data Handling and Statistical Analysis

The data collected was cleaned, validated, and then entered into Microsoft Excel (which was also used to calculate proportions) and then validated prior to descriptive and inferential statistical analyses on Epi Info software. Chi-square (*χ*^2^) test was used to assess significance of association between isolation rates of thermophilic *Campylobacter* spp. and seasons and cattle grazing system. Confidence intervals at 95% level were analysed for proportions, using the Clopper and Pearson exact method using SPSS. *P* value ≤ 0.05 was considered significant.

## 3. Results

### 3.1. Cultural Characteristics

**Figure 2:**
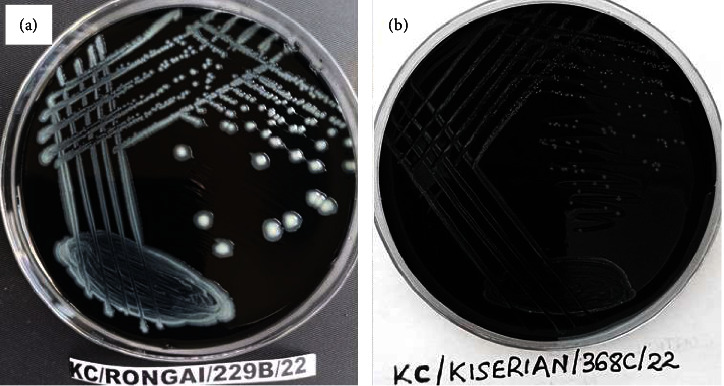
*Campylobacter* colonies on mCCDA plate after 48 hours of microaerobic incubation at 42°C. Medium off-white glistening/shiny and spreading colonies (plate (a)) and the small gray colonies on the media (plate (b)).

The major phenotypic characteristics of the isolates obtained in this study were typical of thermophilic *Campylobacter* species. All isolates demonstrated small-to-medium gray glistening and spreading colonies on mCCDA plates containing selective supplement after 48 hours of microaerobic incubation at 42°C (Figure 2).

**Figure 3:**
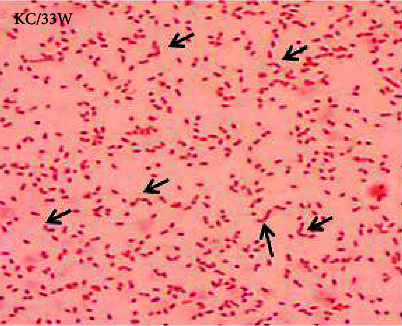
Small Gram-negative short or curved rods to coccobacilli of *Campylobacter* isolate (×1000) with characteristic “seagull” shaped curved rods.

All of the isolates tested exhibited positive oxidase (marked by development of purple colour on oxidase discs within 30 seconds after exposure) and variable catalase reaction (effervescence). The putative *Campylobacter* isolates were further subjected to Gram stain reaction, where they exhibited Gram-negative small curved rods to coccobacilli (Figure 3). Some (48%) suspect *Campylobacter* isolates (putative *C. jejuni* isolates) showed ability to hydrolyze sodium hippurate.

Out of 457 analysed samples, 213 (46.6%) were presumptively positive based on conventional culture-identification-dependent methods (showed characteristic campylobacter colony growth on selective mCCDA plates at 42°C and positive biochemical characteristics) (Table 2). However, a significant proportion of the samples (20.8%, 95/457) were overgrown by noncampylobacter background flora on mCCDA plate with selective supplement. These noncampylobacters were oxidase-negative rods and a subset was confirmed as *E. coli* and *Klebsiella* spp. by matrix-assisted laser desorption/ionisation time-of-flight mass spectrometry (MALDI-TOF-MS). The rest of the samples (149 samples) produced no observable growth after 72 hours of microaerobic incubation at 42°C.

### 3.2. Molecular Detection of *Campylobacter* Genus and Confirmation of *C. jejuni* and *C*. *coli*

**Figure 4:**
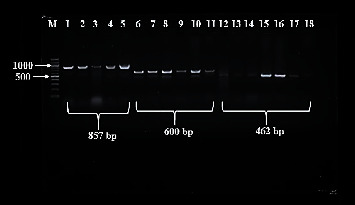
Agarose gel electrophoresis visualisation of PCR amplicons of 857 bp 16S rRNA gene for *Campylobacter* genus (wells 1–5), 600 bp *hipO* gene for *C. jejuni* (wells 6–11), and 462 bp *ceuE* gene for *C. coli* (wells 12–18). M: DNA ladder, where each band represents 100 bp.

Amplification of 857 bp of 16 subunit ribosomal RNA (16S rRNA) gene for identification of *Campylobacter* genus produced bands corresponding to molecular size target as shown in Figure 4.

### 3.3. Overall and Sample-Source Level Prevalence of *Campylobacter* Isolates

Out of the 213 culture positive samples, 162 samples were confirmed as belonging to *Campylobacter* genus by singleplex 16S rRNA PCR, giving an overall sample-level prevalence of 35.4% (95% CI = 31.0–39.8%). The highest prevalence was observed in cloacal swabs of live chicken at 44.4% (63/142, 95% CI = 36.2–52.6%), followed by rectal swabs from live cattle at 30.9% (82/265, 95% CI = 25.3–36.5%). Water samples from cattle drinkers/trough were found to be contaminated at 34% (17/50, 95% CI = 20.9–47.1%). The isolation rate was higher in cattle under confinement system at 44.3% (62/140, 95% CI = 36.1–52.5), than in those under free-roaming grazing system at 16% (20/125, 95% CI = 9.6–22.4%).

### 3.4. Seasonal Prevalence of *Campylobacter* Isolates and Associated Climatic Variables Assessed

Thermophilic *Campylobacter* species were isolated in both seasons, with higher prevalence recorded during rainy and cold season in all sample types except for water (Table 3). There was a significant association between season and thermophilic *Campylobacter* carriage (*χ* = 24.726, *p*=0.000). The cumulative prevalence was higher during the cold-wet/rainy season at 39.8% (98/246, 95% CI = 33.6–45.9), compared to warm-dry season (30.3% (64/211, 95% CI = 24.1–36.5), though the difference was statistically insignificant (*P* value > 0.05).

The mean ± SEM and range of selected continuous climatic variables assessed over the sampling period across the two seasons are shown in Table 4. There were minimal variations (statistically insignificant, *P* value > 0.05) across the seasons, with the lowest temperature, rainfall, and humidity recorded in different seasons at 10.94°C, 0.03 mm, and 42.3%, respectively.

### 3.5. Molecular Confirmation of Genes Coding for *C. jejuni* and *C*. *coli*

Speciation of all culture and 16S rRNA positive isolates by singleplex PCR for *C. jejuni* and *C. coli* produced amplicons corresponding to 600 bp for hippurate hydrolase (*hipO*) and 462 bp for siderophore enterochelin (*ceuE*) genes, respectively (Figure 4).

*Campylobacter jejuni* was the most frequently confirmed thermophilic *Campylobacter* species at 55.6% (95% CI = 47.9–63.3%) in all sample types. 26.5% (95% CI = 19.7–33.3) of the isolates were categorised as other thermophilic *Campylobacter* species (could not be identified further) (Table 5).

## 4. Discussion

Even though *Campylobacter* species are considered commensals in poultry, they have been isolated from cases of bovine abortion, bovine mastitis, enteritis, and/or diarrhoea in calves. In addition, *Campylobacter* poses a public health risk through faecal contamination of milk or drinking water or meat at slaughter. The overall sample-level prevalence of thermophilic *Campylobacter* recorded in this study was 35.4%, whereas prevalence values for chicken, cattle, and water samples were 44.4%, 30.9%, and 34%, respectively. Similar findings were obtained by Uaboi-Egbenni [32] in their study of the prevalence of thermophilic *Campylobacter* species in cattle and chickens in rural areas of Limpopo Province, South Africa. In Kenya, there is paucity of studies on the prevalence of *Campylobacter* from multiple live farm animals and their natural environment (water, soil, or even feeds/pastures) with which to compare the findings of this study. Even though this study's finding is in discrepancy with an earlier study conducted in informal settlements in Nairobi, Kenya, by Chepkwony [7], Chepkwony [7] documented overall prevalence of thermophilic *Campylobacter* species of 21.2% in livestock (with no clear contribution of each of the studied livestock including cattle, goat, pigs, sheep, rabbits, and chicken).

In this study, some colonies on mCCDA plates were overgrown by noncampylobacter organisms, some of which were confirmed to be *Klebsiella* species and *E. coli*. The rate for noncampylobacter flora on this medium was 20.8%. These results indicate that mCCDA medium is not 100% sensitive and selective for isolation of thermophilic campylobacters. A similar finding has been reported by Chon et al. [33] who reported presence and overgrowth of background/nonfastidious flora that interfered with isolation of *Campylobacter*. There is, therefore, a need to enhance selectivity of the culture agar so as to maximise isolation rate. However, these were sorted out when 16S ribosomal RNA typing and speciation PCR were done; some culture positive isolates were not amplified using 16S rRNA or speciation PCR probes for *C. jejuni* and *C. coli.* These were taken to be neither of the two species, even though it is possible that the negative PCR assay could be ascribed to genetic disparities in these isolates, for example, point alterations in the regions complementary to this study's target sequence, thus altering binding by PCR probes and preventing amplification [34]. The quality and quantity of genomic bacterial DNA could also lead to negative PCR results.

Among the 162 16S rRNA PCR positive isolates, 55.6% were confirmed to be *C. jejuni*, followed by *C. coli* (17.9%), and the rest were other thermophilic *Campylobacter* spp. (OTCs; could not be identified). *Campylobacter jejuni* was the most confirmed species in all the sample types (chicken cloacal swabs (66.7%), cattle rectal swabs (51.2%), and trough water samples (35.3%)). The predominance of *C. jejuni* over *C. coli* in all the sample types agrees with previous studies in Kenya and other countries [7,9,13,35–37]. Yet, some studies elsewhere reported *C. coli* as the most confirmed species in bovine [38–40]. Other thermophilic *Campylobacter* species (OTCs) comprise infrequently isolated *Campylobacter* species (*C*. *concisus*, *C. lari*, *C*. *upsaliensis*, and *C*. *ureolyticus)* and have also been reported in both cattle and chicken.

The relatively high *Campylobacter* prevalence in chicken of 44.4% found in this study is comparable to that reported by other studies carried out in Kenya. Poultry are documented asymptomatic reservoirs for thermophilic *Campylobacter*, with studies in Kenya reporting prevalence of 29–44% [9,41]. However, a number of studies in this country have reported prevalence values higher than 44% [8,10,12,35]. Several studies in other Sub-Saharan African countries have reported both lower and higher prevalence values: 69.8% in Tanzania [42], 77.6% in Nigeria [43], 22.5% in Ghana [39], and 28.9% in Ethiopia [44]. Notably, the prevalence of chicken in this study is moderately lower, which might be due to various differences: in isolation techniques, in sampling units (breeds and production systems), or in identification methods. In this study, the cloacal swabs were preenriched in Bolton broth, which has been shown to affect both the number and species of thermophilic *Campylobacter* isolated from naturally contaminated samples [45]. Higher prevalence was observed in chicken compared to cattle. Chicken in smallholder systems are often confined to undesignated houses in the evenings in close interaction with other farm animals, including cattle, where they scavenge for feed leftovers [46]. Subsequently, chicken may play a role in epidemiology of campylobacter infections in cattle.

Thermophilic *Campylobacter* isolates were recovered from 30.9% of the 265 cattle rectal swabs analysed. This finding is consistent with studies conducted in other countries [37,47,48]. There are variations in isolation rate of thermophilic *Campylobacter* in bovine ranging from 4 to 89.4% [49], depending on isolation protocols (direct streaking or enrichment), herd characteristics (age, breed, and production system), seasonal management practices, and sample type (rectal swabs versus dung or gastrointestinal contents). The prevalence reported in this study might reflect where the cattle were sampled, with higher prevalence (44.3%) recorded in zero-grazing/confinement production systems than in outdoor grazing system. However, Grove-White [50] observed higher prevalence among outdoor grazing cattle. A probable explanation to this study's finding is that there is a higher risk of acquiring thermophilic *Campylobacter* from a herdmate (close contact) especially if under group housing and/or under high stocking density. Moreover, in most integrated confined farming systems, there was tendency of spreading slurry (or poorly dried manure) on pastures/fodder crops. Cattle under outdoor grazing system are known to evade grazing on faecal contaminated pasture [51], thus leading to minimal risk of exposure.

Although it is clear that both cattle and chicken are reservoirs, it is likely that water samples collected from water troughs (and/or animal watering points) are a significant *Campylobacter* contaminant at 34%, therefore playing a key role in transmission to livestock. Contaminated water has been incriminated as a significant source of thermophilic *Campylobacter* contagion for bovine [13,20,52,53]. Other thermophilic *Campylobacter* spp. had the highest frequency with *C. jejuni* and *C. coli* appearing less frequently. Similar findings were reported in a study on diverse *Campylobacter* species in water samples from river Bø [54]. Besides *C. jejuni* and *C. coli*, *C. hyointestinalis* and *C. lari* have also been isolated from water [54,55]. Carriage in water samples is an indication of *Campylobacter* contamination rather than colonisation *per se*. However, the carriage may be driven by the ability of the organism to survive outside the host and/or environmental factors, for example, presence of different reservoirs. Therefore, the higher isolation rate for OTCs probably suggests a high degree of contamination involving multiple faecal shedders besides cattle and/or poultry.

Seasonality effect on occurrence of thermophilic *Campylobacter* in Kenya has not been described previously, though a survey by Shimotori et al. [56] reported varying thermophilic *Campylobacter* colonisation in children at 17%, 5.4%, and 12.2% in July (cold and dry), September (hot and dry), and November (wet and cold), respectively. In this study, peaks in prevalence of thermophilic *Campylobacter* spp. in both cattle and chicken were observed during cold and rainy seasons. Yet, Nwankwo et al. [57] reported higher prevalence during cold-dry season (during the months of October–February which are partly rainy and dry in Kajiado) in free range chickens. This study also recorded higher prevalence of thermophilic *Campylobacter* spp. in water samples during the dry and warm period. A probable hypothesis for this finding is that transhumant activities (movement of both livestock and to some extent wildlife migration in search of water and pasture especially during drought) inside and outside the region may contribute to faecal contamination of water especially for natural watercourses or reservoirs (dams). Even though a small seasonal effect (prevalence values differed insignificantly) was observed in this study, the minimal variations may be due to animal husbandry practices, presence of reservoirs (rodents, birds, and flies), or other confounding factors. Indeed, the reported summer and autumn peaks of *Campylobacter* infections in both humans and livestock in Europe and North America are attributable to distinct seasons having discrete climatic conditions. Unlike in Kajiado County where the climatic conditions (ambient temperature, relative humidity, and precipitation) were more or less the same during the two seasons, the small seasonal effect could be attributable to the minimal disparities in climatic variables across the seasons. Kajiado-specific climatic factors that may influence *Campylobacter's* persistence in the environment at the time of sampling include rainfall of 0.03 mm, ambient temperature above 10.94°C, and humidity above 42.3% during the two seasons. In almost comparable findings, Carron et al. [9] suggest that a constant temperature above 16°C or a precipitation of 80 to 191 mm may favour *Campylobacter* survival in the environment. Consequently, this calls for further investigations especially on the biological mechanisms.

## 5. Conclusion

Results of this study demonstrate that cattle, chicken, and water harbour potentially pathogenic thermophilic campylobacters, with higher prevalence observed in chicken. This suggests that cattle and chicken are important reservoirs of *Campylobacter* spp., potentially posing public health hazard. *Campylobacter jejuni* is widely distributed among farms. The isolation rate was higher in cattle under confinement system (44.3%) (95% CI = 36.1–52.5%) than in those under free-roaming grazing system. There were minimal seasonal variations with respect to occurrence of thermophilic *Campylobacter* carriage. *Campylobacter* spp. were isolated in both seasons, with slightly higher prevalence (39.8% (95% CI = 33.6–45.9)) recorded during rainy and cold season in all sample types except for water.

### 5.1. Recommendation

Selective modified charcoal-cefoperazone-deoxycholate agar (mCCDA) has shown poor sensitivity and selectivity for isolation of *Campylobacter*; it is, therefore, recommended to use a more efficient medium for respective isolations. Noncampylobacter (NC) and other thermophilic *Campylobacter* (OTC) isolates should be scrutinised to investigate their role in disease process if any. Further epidemiological and phylogenetic studies comparing livestock, environmental samples, and human thermophilic *Campylobacter* isolates are needed to establish source attribution and zoonotic potential of thermophilic campylobacters.

## Figures and Tables

**Table 1:** Sampling types and distribution per season under different production system.

Sample type	Production system	Seasonal sampling		Total
		Cold and wet season	Dry and warm season	
Cattle rectal swabs	Farm-fed/confined (zero-grazing)	96	44	140
	Free-roaming (outdoor grazing)	24	101	125

Chicken cloacal swabs	Housed	97	45	142

Surface water	Bovine's water troughs and/or watering point	29	21	50

Total		247	211	457

**Table 2:** Summary of culture-based results per individual sample source.

Sample source	Production system	Culture based identification		
		Presumed thermophilic campylobacters (*n*, %)	NC (*n*, %)	No observable growth^*∗∗*^
Cattle rectal swabs (*n* = 265)	Farm-fed/confined (zero-grazing) (*n* = 140)	66 (47.1%)	33 (23.6%)	41 (29.3%)
	Free-roaming (outdoor) grazing systems (*n* = 125)	33 (26.4%)	16 (12.8%)	76 (60.8%)
Chicken cloacal swabs (*n* = 142)	Housed chicken	91 (64.1%)	32 (22.5%)	19 (13.4%)
Surface water sample (*n* = 50)		23 (46.0%)	14 (28.0%)	13 (26.0%)
Total (*n* = 457)		213 (46.6%)	96 (20.8%)	149 (32.6%)

^*∗∗*^Produced no observable growth after 72 hours of microaerobic incubation at 42°C. NC: noncampylobacter (negative for putative *Campylobacter* spp.).

**Table 3:** Seasonality of thermophilic *Campylobacter* isolates from different sample types.

Sample type	Distribution of thermophilic *Campylobacter* isolates			
	Cold-wet (rainy) season		Warm-dry season	
	% (n/N)	95% CI	% (n/N)	95% CI
Cattle rectal swabs	40.8% (49/120)	32.0–49.6	22.8% (33/145)	16.0–29.6
Chicken cloacal swabs	46.4% (45/97)	36.5–56.3	40.0% (18/45)	25.7–54.3
Surface water samples	13.8% (4/29)	1.2–26.4	61.9% (13/21)	41.1–82.7
Total	39.8% (98/246)	33.6–45.9	30.3% (64/211)	24.1–36.5

**Table 4:** Mean ± SEM and range of selected climatic variables collected during field survey conducted from October 2021 to May 2022 in Kajiado County.

Climatic variables	Warm-dry season		Wet-rainy season	
	Mean ± SEM	Range	Mean ± SEM	Range
Average rainfall amount (mm)	11.22 ± 3.35	0.03–53.60	76.47 ± 16.16	8.4–305.4
Daily maximum temperature (°C)	23.35 ± 0.62	19.39–27.57	24.41 ± 0.33	19.97–25.2
Daily minimum temperature (°C)	12.93 ± 0.35	10.94–15.42	14.73 ± 0.26	11.04–14.83
Relative humidity (%)	55.86 ± 2.22	47.68–65.00	54.52 ± 2.20	42.30–69.57

**Table 5:** Molecular typing of thermophilic *Campylobacter* species across sample types.

Sample type	Number analysed by PCR	PCR positive (N)	Species distribution of *Campylobacter* isolates					
			*C. jejuni*		*C. coli*		OTCs	
			%	95% CI	%	95% CI	%	95% CI
Cattle faeces	100	82	51.2%	40.4–62.0	19.5%	10.9–28.1	29.3%	19.4–39.2
Chicken cloacal swabs	91	63	66.7%	55.1–78.3	14.3%	5.7–22.9	19.0%	9.3–28.7
Drinking/trough water	22	17	35.3%	12.6–58.0	23.5%	3.3–43.7	41.2%	17.8–64.6
Total	213	162	55.6%	47.9–63.3	17.9%	12.0–23.8	26.5%	19.7–33.3

OTCs: other thermophilic *Campylobacter* spp. that were not identified; 95% CI: 95% confidence intervals for the proportions.

## Data Availability

The data that support the findings of this study are available from the corresponding author upon request.
